# Metrics for GO based protein semantic similarity: a systematic evaluation

**DOI:** 10.1186/1471-2105-9-S5-S4

**Published:** 2008-04-29

**Authors:** Catia Pesquita, Daniel Faria, Hugo Bastos, António EN Ferreira, André O Falcão, Francisco M Couto

**Affiliations:** 1 XLDB, Departamento de Informática, Faculdade de Ciências da Universidade de Lisboa, Campo Grande - Edifício C6, Lisboa, Portugal; 2 Centro de Química e Bioquímica, Departmento de Química e Bioquimica, Faculdade de Ciências da Universidade de Lisboa, Campo Grande - Edificio C8, Lisboa, Portugal

## Abstract

**Background:**

Several semantic similarity measures have been applied to gene products annotated with Gene Ontology terms, providing a basis for their functional comparison. However, it is still unclear which is the best approach to semantic similarity in this context, since there is no conclusive evaluation of the various measures. Another issue, is whether electronic annotations should or not be used in semantic similarity calculations.

**Results:**

We conducted a systematic evaluation of GO-based semantic similarity measures using the relationship with sequence similarity as a means to quantify their performance, and assessed the influence of electronic annotations by testing the measures in the presence and absence of these annotations. We verified that the relationship between semantic and sequence similarity is not linear, but can be well approximated by a rescaled Normal cumulative distribution function. Given that the majority of the semantic similarity measures capture an identical behaviour, but differ in resolution, we used the latter as the main criterion of evaluation.

**Conclusions:**

This work has provided a basis for the comparison of several semantic similarity measures, and can aid researchers in choosing the most adequate measure for their work. We have found that the hybrid *simGIC* was the measure with the best overall performance, followed by Resnik's measure using a best-match average combination approach. We have also found that the average and maximum combination approaches are problematic since both are inherently influenced by the number of terms being combined. We suspect that there may be a direct influence of data circularity in the behaviour of the results including electronic annotations, as a result of functional inference from sequence similarity.

## Background

One of the main contributions of bioinformatics in molecular biology has been the introduction of ontologies for genome annotation. These circumvent the shortcomings of natural language descriptions (namely ambiguity, subjectivity and lack of structure) and consequently enable automated annotation and automated reasoning over annotations [1]. Prominent among these is the Gene Ontology (GO), which is dedicated to the functional annotation of gene products in a cellular context and a species independent manner [2]. It comprises three orthogonal ontologies (GO types) organised as directed acyclic graphs (DAGs) which account for distinct aspects of gene products: *molecular function*, *biological process* and *cellular location*. The relationships between GO terms can be either *is-a* (parent-child) or *part-of* (part-whole) relationships.

Among other applications, the use of ontologies such as GO enables the comparison of gene products based on their annotations, so that functional relationships and common characteristics can be inferred beyond the traditional sequence-based approaches. This requires the use of a semantic similarity measure to compare the terms to which gene products are annotated. There are two main approaches to measure semantic similarity [1]: edge-based measures, which assume a term's specificity can be directly inferred from its depth in the graph; and information content (IC)-based measures, which estimate a term's specificity from its usage frequency within a given corpus. In the case of GO (as in many other biological ontologies) the latter are more adequate because specificity is poorly related with depth in the graph, for instance: the terms *binding* and *translation regulator activity* are at the same depth but the latter is both semantically more complex and biologically more specific.

Lord et al. [3,4] were the first to apply GO-based semantic similarity to compare gene products, testing three IC-based measures: Resnik's [5], Lin's [6], and Jiang and Conrath's [7]. These three measures, originally developed for WordNet, compare terms by finding their lowest common ancestor (LCA). However, the definition of LCA is not straightforward in GO, since GO terms can have several disjoint common ancestors. Lord et al. [3,4] addressed this issue by using only the most informative common ancestor (MICA), while later, Couto et al. [8] considered that all disjoint common ancestors should be taken into account.

A more critical issue when applying these measures to gene products is that they are measures for comparing single terms whereas gene products have usually several terms (within each GO type). Therefore, obtaining a single similarity score requires combining the semantic similarities of the gene products' terms (of the same GO type). Three distinct approaches have been proposed for this combination: Lord et al. [3,4] used an arithmetic average of the term similarities, pairing all terms of the first gene product with all terms of the second one; Sevilla et al. [9] used only the maximum similarity between all term pairs; and Couto et al. [10], Schlicker et al. [11] and Azuaje et al. [12] developed composite (best-match) averages where each term of the first gene product is paired only with the most similar term of the second one and vice-versa.

From a biological point of view, there are limitations to both the average and maximum approaches. The average approach is inaccurate for gene products with several shared or similar terms, for instance: two functionally identical gene products having both the terms *antioxidant activity* and *binding* have a similarity of 50% rather than the expected 100%, because similarities are calculated between all possible term pairs of the two gene products. By contrast, the maximum approach is indifferent to the number of unrelated terms between gene products, for instance: a gene product with the terms *antioxidant activity* and *binding* and a second gene product with only one of those terms would have a similarity of 100%, when functionally they are clearly not equal. The best-match average approach does not suffer from the above limitations, and accounts for both similar and dissimilar terms as would be expected biologically.

A different approach to the issue of gene products having more than one term (within each GO type) is to use a semantic similarity measure that compares sets of terms rather than single terms, thus avoiding the need to combine similarities. Since the set of GO terms of a given type to which a gene product is annotated can be seen as a sub-graph of that GO type, a graph comparison measure can be used for this purpose. Gentleman [13] was the first to explore this possibility by developing the *simUI* measure, which given the annotation graphs for two gene products, defines semantic similarity as the fraction between the number of GO terms in the intersection of those graphs and the number of GO terms in their union. Despite accounting for both similar and dissimilar terms in a simpler way than finding matching term pairs, this measure weights all terms equally, and therefore does not account for term specificity. To overcome this limitation, we developed the *simGIC* measure, which is similar to *simUI*, but where each term is weighted by its information content [14].

Applications of GO-based semantic similarity have been innumerous, and include such diverse subjects as: protein interaction prediction [15], validation of function prediction [16], network prediction [17], prediction of cellular localisation [18], automatic annotation validation [19], integration of semantic search [20], pathway modelling [21], and improving microarray data quality [22]. However, two crucial questions still stand: which type(s) of annotations should be trusted for semantic similarity calculations; and which semantic similarity measure performs better with GO?

The first question is central to current molecular biology. On one hand it has become clear with the advent of automated sequencing that experimental work cannot be the sole source for gene product annotation, if the gap between sequence data and functional information is to be bridged. On the other hand, the increasingly important role for bioinformatics in annotation [23] has lead to a growing number of annotations extrapolated from sequence similarity, which are prone to errors [24,25]. Indeed, it has been suggested that as much as 30% of the annotations corresponding to detailed characteristics can be erroneous as a result of inferring annotations from sequence similarity, particularly from gene products whose annotations had already been extrapolated [26,27]. Despite this, the precision of automated annotations methods has been increasing steadily (up to 91-100% reported [28]), and as they account for a growing portion of the annotation space (currently over 97% of all Uniprot [29] GO annotations), the cost of ignoring them becomes heavier.

As for which semantic similarity measure is more suitable to GO, it raises another question: how do you evaluate the performance of semantic similarity measures? Authors have used correlations with sequence similarity [3,4], with Pfam similarity [10], with gene co-expression [9], and with protein interactions [21] to evaluate their measures; some discarding electronic (and other) annotations [3,4,11] and others using all annotations [9]. This profusion of evaluation strategies, with new results not being directly comparable to previous ones, hinders the extraction of any global conclusion about the measures' performances.

In this work, we perform a systematic evaluation of several semantic similarity measures. The mould of this evaluation was to assess, given a set of gene products and a corpus of GO annotations, how well each semantic similarity measure captures the similarity in annotations between gene products. As there is no internal means of making this assessment, an external source of data, correlated with the annotations, must be used. We opted for using sequence similarity, since it is well established to be related to function and there is some insight on that relation, namely: general functional characteristics are conserved at relatively low levels of sequence similarity (30%), while specific functional characteristics are poorly conserved even at high levels (70%) [24]. Since we have this type of insight only between function and sequence, and because the other GO types have been shown to have a looser correlation with sequence similarity [3,4], only the *molecular function* GO type was used. To summarise our strategy, we are evaluating the measures by assessing how well they capture the expected relationship between functional and sequence similarity.

## Results and discussion

To evaluate the semantic similarity measures, we used two distinct sequence similarity measures: log reciprocal BLAST score (LRBS) and relative reciprocal BLAST score (RRBS). The former is similar to the sequence similarity measure used previously by Lord [3,4], but compensates for the fact that BLAST scores are not symmetric, while the latter is analogous to the sequence identity percentage (which has recently been suggested as a good indicator of functional similarity [25]) but takes amino acid substitutions into account. As RRBS is not directly affected by sequence length (unlike LRBS) we can assess whether the dependency on sequence length affects the outcome of the evaluation.

A total of fourteen semantic similarity measures were tested: Resnik's, Lin's, and Jiang and Conrath's term similarity measures, each with the average, maximum, best-match average (BMA), and BMA plus GraSM approaches; plus the graph-based simUI [13] and simGIC measures [14]. We evaluated the influence of using electronic annotations by testing the measures on two distinct datasets: one with all annotations (full dataset) and one without electronic annotations (non-electronic dataset).

### Modelling the behaviour of semantic similarity

The raw semantic similarity *vs*. sequence similarity results were averaged over intervals of fixed number of points (as detailed in the Methods section), so that the global behaviour of the results could be perceived.

**Figure 1:**
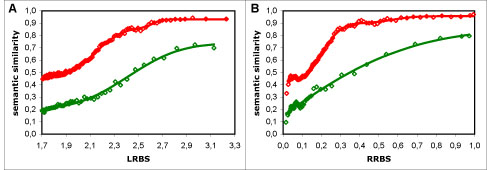
**Typical behaviour of semantic similarity measures.** Semantic similarity *vs*. sequence similarity results using Resnik's measure with the BMA approach: A - using the LRBS sequence similarity metric; B - using the RRBS metric; in red - full dataset results (points) and modelling curve (line) composed of two additive normal cumulative distribution functions; in green - non-electronic dataset results (points) and modelling curve (line) composed of a single normal cumulative distribution function. The results for the full dataset show a bimodal-like behaviour: there is a second increase in semantic similarity after a first plateau has been reached, which is more pronounced in A, but also visible in B. This behaviour is absent in the non-electronic dataset.

Upon observing the averaged results, it was clear that their behaviour was not linear (as is visible in Figure 1), regardless of dataset, sequence similarity measure or semantic similarity measure used. What is more, within each dataset and sequence similarity measure, the majority of the semantic similarity measures were similar in behaviour, showing the same patterns in regard to sequence similarity. Therefore, it was necessary to find a type of function that followed the overall behaviour of the results closely, and that could be fitted to all semantic similarity measures (for a given dataset and sequence similarity measure) so that we could quantify the differences between them. We chose to use rescaled Normal cumulative distribution functions (*N_CDF_*s), which correspond to error functions, because in addition to fulfilling this requirement, the influence of their parameters in the shape of resulting curve is intuitive, and they promote a simple probabilistic interpretation of our results. The results from the full dataset, where a bimodal-like behaviour was evident (visible as a second increase in semantic similarity after a first plateau had been reached), were modelled by two additive *N_CDF_*s, whereas those from the non-electronic dataset required only a single *N_CDF_* (Figure 1); in both cases, scale (multiplicative) and translation (additive) parameters were applied to fit the range of the results (as detailed in the Methods section).

Confirming their visible similarity, the majority of the semantic similarity measures (the exceptions will be addressed individually in the case-by-case discussion) have modelling functions with identical shape parameters (mean and standard deviation of the *N_CDF_*), and differ mostly in range (Tables 1 and 2). This means that the majority of the measures capture the same pattern (or variations) along the sequence, but tend to translate that pattern into different ranges of the semantic similarity scale. It should be noted that this difference in range occurs only in the averaged semantic similarity results, and not between the actual semantic similarity measures, which are all ranged in a 0-1 scale (and the whole range of that scale is covered by the raw results). Therefore, the range of the results should be interpreted as a tendency of the measure, rather than a scale limit. This tendency is composed by two distinct properties: bias, *i.e*. the tendency to yield higher semantic similarity values, which is measured by the translation parameter of the modelling function; and resolution, *i.e* the relative intensity with which (on average) variations in the sequence similarity scale are translated into the semantic similarity scale, which is measured by the scale parameter of the modelling function (or the sum of the two scale parameters in bimodal functions). A measure with a higher bias than another will likely yield a higher value of semantic similarity for a given value of sequence similarity, whereas a measure with a higher resolution than another will likely yield a greater variation in semantic similarity for a given interval of sequence similarity.

**Table 1:** Parameters of the modelling functions for the full dataset

		LRBS					RRBS				

		Mean1	Stdev1	Mean2	Stdev2	Res.	Mean1	Stdev1	Mean2	Stdev2	Res.
simGIC		2,1	0,20	2,6	0,06	**0,62**	0,21	0,07	0,58	0,10	**0,65**
simUI		2,1	0,20	2,6	0,05	0,43	0,21	0,08	0,59	0,07	0,46

Resnik's measure	Avg	2,1	0,28	2,7	0,08	0,16	0,21	0,10	0,49	0,03	0,35
	Max	2,1	0,05	2,6	0,18	0,24	0,16	0,06	0,49	0,22	0,37
	BMA	2,1	0,18	2,6	0,06	0,47	0,20	0,08	0,58	0,09	0,55
	GraSM	2,2	0,19	2,6	0,03	**0,59**	0,23	0,08	0,50	0,01	**0,67**

Lin's measure	Avg	2,1	0,31	2,7	0,08	0,15	0,21	0,12	0,49	0,03	0,29
	Max	2,1	0,08	2,6	0,15	0,18	0,15	0,08	0,49	0,15	0,30
	BMA	2,1	0,20	2,6	0,06	0,39	0,20	0,08	0,33	0,34	0,47
	GraSM	2,2	0,18	2,6	0,03	0,47	0,22	0,09	0,50	0,01	0,57

Jiang & Conrath's measure	Avg	2,1	0,24	2,7	0,06	0,10	0,19	0,10	0,49	0,03	0,20
	Max	2,1	0,04	2,4	0,43	0,14	0,16	0,08	0,49	0,15	0,21
	BMA	2,1	0,16	2,6	0,09	0,22	0,19	0,08	0,45	0,25	0,28
	GraSM	2,2	0,16	2,6	0,03	0,27	0,20	0,10	0,51	0,00	0,38

For each semantic similarity measure in the full dataset, and with each of the sequence similarity metrics (LRBS and RRBS), the mean and standard deviation parameters for the two additive normal cumulative distribution functions (*N_CDF_*) used to model it are shown. Also shown is the global resolution of the measure, which corresponds to the sum of the scale factors applied to each of the normal functions. Although there is some variability on the normal parameters (particularly in the results with the RRBS sequence similarity metric), most of that variability is due to the sensitivity of the modelling method, as the similarity in behaviour between the measures is evident (Figures 3, 4 and 5) with the exception of the average approach. As the main criterion to distinguishing between the measures is their resolution, the highest resolutions (for simGIC and Resnik's measure with the GraSM approach) are highlighted in bold (Mean1: mean of the first *N_CDF_*; Stdev1: standard deviation of the first *N_CDF_*; Mean2:mean of the second *N_CDF_*; Stdev2: standard deviation of the second *N_CDF_*; Res: resolution of each measure; LRBS: log reciprocal BLAST score; RRBS: relative reciprocal BLAST score).

**Table 2:** Parameters of the modelling functions for the non-electronic dataset

		LRBS			RRBS		

		Mean	Stdev	Res.	Mean	Stdev	Res.
simGIC		2,4	0,33	**0,58**	0,25	0,32	**0,65**
simUI		2,4	0,35	0,45	0,29	0,30	0,50

Resnik's measure	Avg	2,3	0,31	0,34	−1,05	0,77	0,46
	Max	2,4	0,34	**0,58**	0,08	0,41	**0,64**
	BMA	2,4	0,34	**0,54**	−0,11	0,58	**0,64**
	GraSM	2,4	0,30	0,40	−0,53	1,16	0,56

Lin's measure	Avg	2,3	0,33	0,32	−1,24	0,78	0,42
	Max	2,4	0,35	0,50	0,18	0,34	0,54
	BMA	2,4	0,35	0,49	0,01	0,51	0,57
	GraSM	2,4	0,31	0,40	0,14	0,60	0,54

Jiang & Conrath's measure	Avg	2,3	0,33	0,21	0,25	0,20	0,24
	Max	2,4	0,33	0,29	0,32	0,17	0,29
	BMA	2,4	0,33	0,29	0,32	0,21	0,32
	GraSM	2,4	0,30	0,31	0,39	0,27	0,39

For each semantic similarity measure in the non-electronic dataset, and with each of the sequence similarity metrics (LRBS and RRBS), the mean and standard deviation parameters for the normal cumulative distribution function used to model it are shown, as well as the global resolution of the measure. The variability on the normal parameters with RRBS is evident because the fit is somewhat artificial, and does not reflect the fact that the behaviour of measures is visibly isomorphic. The highest resolutions, corresponding to simGIC and Resnik's measure with the maximum and BMA approaches, are highlighted in bold (Mean: mean of the *N_CDF_*; Stdev: standard deviation of the *N_CDF_*; Res: resolution of each measure; LRBS: log reciprocal BLAST score; RRBS: relative reciprocal BLAST score).

The goal of our evaluation was to assess how well each semantic similarity measure captures the similarity in annotation between protein pairs. While previous studies have made this type of assessment by measuring linear correlation [3,9,10], or analysing a ROC (receiver operating characteristic) curve [21], neither approach is suitable for our results because they are neither linear in behaviour nor binary in nature. Since the majority of the measures show identical behaviours, we focused on the differences between them with implications on their performance, and choose resolution as an evaluation criterion. A measure with a higher resolution performs better because it is more likely to distinguish between protein pairs with different levels of sequence similarity than a measure with a lower resolution, which suggests it is more sensitive to differences in the annotations.

### Full vs. non-electronic dataset

Overall there are two main differences between the results from the full dataset and those from the non-electronic dataset (as can be seen in Figure 1): semantic similarity values are globally lower in the latter than in the former; and the bimodal-like behaviour evident in the former is absent in the latter.

**Figure 2:**
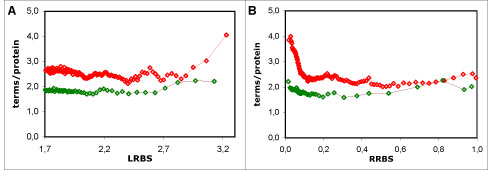
**Distribution of the average number of GO term annotations per protein**. Average number of GO term annotations per protein as function of sequence of sequence similarity: A - using the LRBS sequence similarity metric; B - using the RRBS metric; in red - full dataset; in green - non-electronic dataset. Globally, the number of annotations per protein is higher and less uniform in the full dataset than in the non-electronic dataset. There is a visible increase in annotations per protein for high LRBS values in the full dataset, and also a visible decrease for low RRBS values in both datasets.

The lower semantic similarity values can be explained by the fact that the number of annotations per protein is smaller in the non-electronic than in the full dataset (Figure 2). Because the proteins have less terms, they are less likely to have shared or similar terms, and therefore have lower semantic similarity.

As for the bimodal-like behaviour in the full dataset, we hypothesise that it is a direct result of data circularity, due to the presence of functional annotations inferred from sequence similarity within the electronic annotations. Because functional inference is predominantly made at high levels of sequence similarity, if there was a visible influence of data circularity in our results, we would expect it to be in the form of an abnormal increase in semantic similarity from a given point of the sequence similarity scale. Therefore, the hypothesis of data circularity is consistent with the observed second increase in semantic similarity at high sequence similarity values in the full dataset and with the absence of that behaviour in the non-electronic dataset (Figure 1).

We also considered the possibility that this behaviour was tied to the distribution of the number of annotations per protein, as there is a peak of annotations per protein consistent with the range of the transition between “modes” for the LRBS results (Figure 2). However, the absence of a corresponding pattern for the RRBS results is an argument against this possibility.

It should be noted that whether or not the bimodal-like behaviour is a consequence of data circularity doesn't affect the validity of our evaluation. While the issue of data circularity can be of dire consequences when applying semantic similarity for specific purposes, our evaluation of the semantic similarity measures is in no way based on the assumption that all annotations are correct. We are only assuming that there is a relationship between the annotations and sequence similarity (be it artificially reinforced by data circularity or not) and testing how well each semantic similarity measure captures that relationship.

### LRBS vs. RRBS

The differences in the results using the two sequence similarity measures (LRBS and RRBS) can be divided into two categories: shape differences, as reflected by the mean and standard deviation parameters of the modelling *N_CDF_*s; and range differences, as reflected by the translation and scale parameters (Tables 1 and 2). The shape differences correspond to differences in the sequence similarity scale, and are likely due to the fact that LRBS is a logarithmic measure whereas RRBS is a linear measure. Indeed, we verified that upon rescaling either sequence similarity measure to the scale of the other one, the results from both measures are described by *N_CDF_*s with identical shape parameters (data not shown). As for the range differences, they are likely tied to the other key difference between the measures: the fact that LRBS is biased by sequence length and RRBS is not. Because of this difference, an increase in RRBS corresponds only to an increase in “actual” sequence similarity, whereas an increase in LRBS can be partially due to an increase in sequence length. Therefore, we would expect semantic similarity to be more strongly related with RRBS than with LRBS, assuming there is no direct correlation between semantic similarity and sequence length. Consistent with this hypothesis, we find that for all measures tested, the resolution is higher with RRBS than with LRBS.

The influence of the bias for sequence length is also visible in the distribuition of the average number of annotations per protein (Figure 2): there is a clear increase in annotations per protein at high LRBS values, whereas there is a sharp decrease in annotations per protein at low RRBS values. These differences can be due to the presence of large bifunctional proteins, which are expected to have a greater number of terms (note that each functional aspect of a protein is typically described by several terms). The fraction of these large proteins in each averaged data point is expected to increase with the LRBS scale, which would account for the increasing number of annotations per protein for high LRBS values. Furthermore, alignments between large proteins of low sequence identity, will yield relatively high LRBS values, but low RRBS values. The presence of these alignments is likely more predominant at lower RRBS values, which accounts for the higher number of annotations per protein for those values.

It should be noted that in the case of the RRBS results with the non-electronic dataset the parameters of the modelling functions are not identical between semantic similarity measures (Table 2). This happens because these results do not match the typical *N_CDF_* shape (*e.g*. they have no apparent inflexion point in the majority of the cases), and therefore the mean and standard deviation are not restrained to the range of the results. Because of this, the resolution of the measures could not be obtained from the modelling function and was instead calculated directly from the results (as detailed in the Methods section). However, after re-scaling them to the LRBS scale, all results followed a *N_CDF_* curve with identical mean and standard deviation (data not shown), leading to the conclusion that the differences in shape in the RRBS scale were only apparent.

### Combining term similarity measures

**Figure 3:**
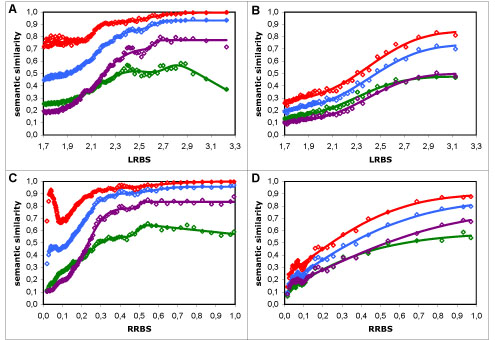
**Comparison of four approaches to term similarity measures**. Semantic similarity *vs*. sequence similarity results using four distinct approaches to Resnik's measure: maximum (in red), average (in green), BMA (in blue) and BMA + GraSM (in violet). A - in the full dataset with the LRBS sequence similarity metric; B - in the non-electronic dataset with the LRBS metric; C - in the full dataset with the RRBS metric; D - in the non-electronic dataset with the RRBS metric. Modelling curves in A and C were composed of two additive normal cumulative distribution functions, and the curve for the average included also a negative linear component; in B and D, all curves were composed of a single normal function. It is noticeable that while all four approaches exhibit similar behaviour in the non-electronic dataset (B and D), the maximum and particularly the average approach perform poorly in the full dataset (A and C), with the former having a very low resolution and the latter showing a decreasing behaviour for high sequence similarity values. The same behaviours and the same relationships between the approaches were obtained for Lin's and Jiang and Conrath's measures.

**Figure 4:**
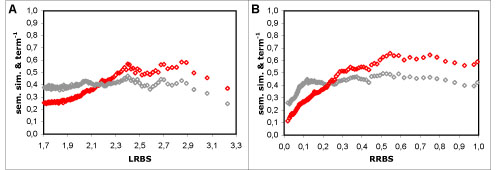
**Relation between the average approach and the inverse of the number of annotations per protein**. Resnik's term similarity measure with the average combination approach (in red) and inverse of the number of annotations per protein (in grey) as function of sequence similarity: A - using the LRBS sequence similarity metric; B - using the RRBS metric. There is an evident parallel between the behaviour of the semantic similarity results and the distribution of the inverse of the number of annotations per protein, which becomes more evident for high sequence similarity values. This parallel reflects the inverse proportionality relationship between the average combination approach and the number of annotations per protein.

In the full dataset, the average combination approach differs from all other measures and approaches tested in that it shows a decreasing behaviour for high sequence similarity values (Figure 3). In order to describe this behaviour, the modelling function for these results required the addition of a negative linear component to be suitably modelled (as detailed in the Methods section). As we clearly do not expect functional similarity and sequence similarity to be negatively correlated, and this behaviour is exclusive to the average approach, we can only infer that this approach is unable to capture the actual similarity in annotations for proteins with high sequence similarity. The reason behind this behaviour is likely tied with the limitations of the average approach, namely to the fact that it considers proteins as random collections of features. For instance, if two proteins (*A* and *B*) have the exact same two terms (*t*1 and *t*2), the average approach compares not only the matching term pairs (*t*1_*A*_ with *t*1_*B*_ and *t*2_*A*_ with *t*2_*B*_) but also all the unrelated ones (*t*1_*A*_ with *t*2_*B*_ and *t*2_*A*_ with *t*1_*B*_). The consequence of this is that the more terms two functionally identical (or similar) proteins have, the less similar they will be considered by the average approach. Consistent with this notion, we find that in the range of values where the average approach shows a decreasing behaviour, there is an inversely proportional increase in the average number of annotations per protein as function of sequence similarity (Figure 4). Indeed, for high sequence similarity values, the behaviour of the average results is deeply tied with the inverse of the number of annotations per protein. Curiously, we have found that if the results with the average approach are compensated for number of annotations per protein, their behaviour becomes identical to that of the other measures (results not shown).

While in the non-electronic dataset the average approach is similar in behaviour to the other approaches (Figure 3), this is likely because overall the number of annotations per protein is smaller in this dataset and also because it is more uniform over the sequence similarity scale (Figure 2). Despite this, the average approach is also the worst combination approach in this dataset, as it shows the lowest resolution (Table 2).

As for the maximum approach in the full dataset, its low resolution (Table 1) is a consequence of its simplicity. Because this approach only looks for the most similar terms between two proteins, it is impervious to the number and similarity of other terms those proteins might have; therefore it is naturally limited in its ability to distinguish protein pairs. In addition, the maximum approach also shows singular behaviours at low sequence similarity values: with the LRBS measure it shows high dispersion, whereas with the RRBS measure it shows a decreasing behaviour (Figure 3). Interestingly, both behaviours are directly related to the distribution of the average number of annotations per protein (Figure 2). This is not unexpected, since the more terms two unrelated (or distantly related) proteins have, the more probable it is that they have a common (or similar) term, and therefore the higher their semantic similarity will be with the maximum approach. In the non-electronic dataset, the limitations of the maximum approach are not visible because the number of annotations per protein is lower in this dataset, with the majority of the proteins having only one annotation (data not shown). Therefore the loss of information from using only one term to compare proteins is negligible, which is why this approach is similar in resolution to the BMA approach.

The BMA approach is clearly the best combination approach in the full dataset, since it not only yields the highest resolutions, but also also does not show the undesired behaviours of the other two approaches. This is because this approach considers all terms of the proteins (and so there is no loss of information), but compares only each term with its most similar (and so is not biased by the number of annotations per protein). Its performance is similar to the maximum approach in the non-electronic dataset, because the number of annotations per protein is small, and therefore there is not much term similarity combination involved.

In conclusion, the average approach is contradictory with the purpose of combining term similarities, due to its dependency on the number of annotations per protein; the maximum approach is limited in its ability to compare proteins as it looks for only one shared functional aspect; whereas the BMA approach is able to account for all functional aspects independently of the number of annotations per protein.

### The influence of GraSM

Compared to the most informative common ancestor (MICA) approach [3,4], the GraSM approach [10] produced systematically lower semantic similarity values (*i.e*. a decrease in the bias of the measures), regardless of dataset or sequence similarity measure (Figure 3). This is a natural consequence of this approach: since it considers the average information content (IC) of all disjoint common ancestors instead of only the IC of the MICA, it will necessarily yield smaller or equal semantic similarity values (equal only if all disjoint common ancestors have the same IC, or there is only one disjoint common ancestor). However, the main question is whether considering more of the GO graph's information (as does GraSM) increases the performance of the semantic similarity measures. In the full dataset, the answer to this question is positive, as GraSM leads to an increase in resolution (20-36%) for all measures tested (Table 1); but in the non-electronic dataset the results are not conclusive, as GraSM increases the resolution of Jiang and Conrath's measure, but decreases that of Lin's and Resnik's measures (Table 2).

### Resnik's, Lin's and Jiang & Conrath's measures

**Figure 5:**
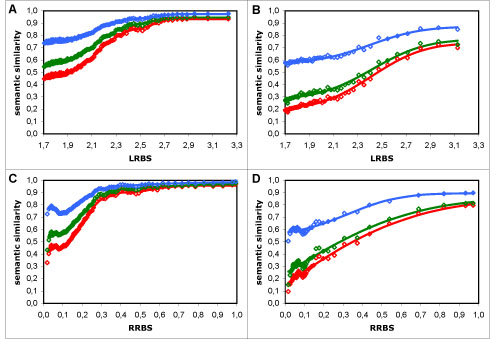
**Comparison of the three term similarity measures.** Semantic similarity *vs*. sequence similarity results using Resnik's (in red), Lin's (in green), and Jiang and Conrath's (in blue) measures with the BMA approach: A - in the full dataset with the LRBS sequence similarity metric; B - in the non-electronic dataset with the LRBS metric; C - in the full dataset with the RRBS metric; D - in the non-electronic dataset with the RRBS metric. These results show that the absolute semantic similarity values increase but the resolution decreases from Resnik's to Lin's to Jiang and Conrath's measures. The same relationship was observed using the maximum, average, and GraSM approaches.

Independently of approach, dataset or sequence similarity metric, the relationship between Resnik's, Lin's, and Jiang and Conrath's measures is always the same (Figure 5): Resnik's measure has the lowest bias and the highest resolution; Jiang and Conrath's measure has the highest bias and the smallest resolution; and Lin's measure falls in between the two (Tables 1 and 2). This relationship is obviously tied to the measures' definitions of semantic similarity: Resnik's measure is directly given by the IC of the MICA of two terms; Lin's measure is given by a ratio of ICs; and Jiang and Conrath's measure is given by a subtraction of ICs. Therefore, while all three measures produce results in the same range (0-1), they behave differently within that range, which leads to their different resolutions. We can only conclude that Resnik's measure is the term similarity measure most adequate for GO, since it consistently shows the highest resolution.

### simGIC and simUI

**Figure 6:**
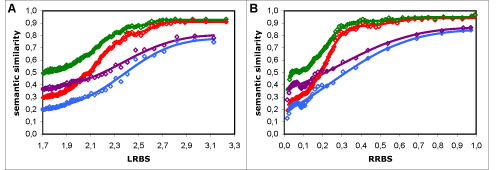
**simGIC and simUI measures**. Semantic similarity *vs*. sequence similarity results using the simGIC and simUI measures: in red - simGIC in the full dataset; in green - simUI in the full dataset; in blue - simGIC in the non-electronic dataset; in violet - simUI in the non-electronic dataset; A - with the LRBS sequence similarity metric; B - with the RRBS metric. Both measures show similar behaviours to those of the term measures with the BMA approach, with simGIC having a higher resolution than simUI and indeed the highest overall resolution of all measures tested.

The graph-based *simUI* and *simGIC* measures showed an identical behaviour to that of the term similarity measures combined with the BMA approach, suggesting that qualitatively both graph-based and term-based approaches are suitable for protein semantic similarity (Figure 6). However, the fact that *simGIC* showed the overall highest resolutions suggests that quantitatively there is an advantage in considering the information conveyed by the structure of the GO graph, rather than just individual annotations. Furthermore, *simUI* and *simGIC* have the clear advantage of being computed in a single step, without the need to find matching terms, and independently of the number of annotations per protein.

From the relationship between *simUI* and *simGIC*, we can conclude that, while GO-based semantic similarity can be accurately measured without IC, using it considerably improves the resolution of the measure (since *simGIC* is a hybrid measure that uses IC in addition to graph structure while *simUI* does not).

From all measures and approaches tested, we conclude that the *simGIC* is the best suited to measure protein semantic similarity, as it yields the highest overall resolutions, which reflects a greater sensitivity to differences in annotation.

## Conclusions

Due to the number of GO-based semantic similarity measures proposed over recent years, and to the diversity of strategies used to evaluate them, the questions of which measure performs better and what are the advantages and limitations of each measure were still open. To tackle these questions, we compared the majority of the existing GO-based semantic similarity measures, and evaluated their performance by assessing how well they capture the expected relationship between functional similarity (as described by *molecular function* GO terms) and sequence similarity. The influence of electronic annotations was assessed by using two separate datasets, while the effect of protein sequence length was investigated by using two distinct sequence similarity metrics.

For all measures tested, we found that the relationship captured between functional and sequence similarity is not linear. The majority of the measures were similar in behaviour, and could be suitably modelled by rescaled Normal cumulative distribution functions with the same shape parameters (mean and standard deviation). One of the key differences between the measures was their resolution, *i.e* the relative intensity with which variations in the sequence similarity scale are translated into the semantic similarity scale. This was the main criterion used to evaluate the measures since it reflects their sensitivity in capturing the relationship between semantic similarity and sequence similarity.

Of the three term similarity measures tested, Resnik's measure was the best, having consistently a higher resolution than Lin's and Jiang and Conrath's measures. As for the approaches to combine term similarities, the best-match average approach was clearly the best, not only because it had the overall highest resolutions, but also because it is independent of the number of terms being combined, unlike the average and maximum approaches. The GraSM approach significantly increased the resolution of the measures in the full dataset (20-33%), but produced inconclusive results in the non-electronic dataset. The *simGIC* measure was overall the best performing measure, showing consistently a high resolution. By comparing the *simGIC* and *simUI* measures, we conclude that while the use of the information content in a measure is not essential to accurately convey semantic similarity, it significantly increases its resolution (19-44%).

We suspect that there may be an influence of data circularity in the results for the full dataset, as the bimodal-like behaviour in this dataset is consistent with the inference of functional annotations between proteins of relatively high sequence similarity. The absence of bimodality in the non-electronic dataset suggests that the effect of data circularity is mainly due to the presence of electronic annotations.

The other major differences between the two datasets are the number of proteins and the number of annotations per protein, which are considerably smaller in the non-electronic dataset as a result of discarding electronic annotations. This loss of information is perhaps the best support for the use of all annotations in large-scale studies, whereas in specific applications where annotation quality is crucial, the use of electronic annotations should be carefully considered. However, as electronic annotations grow in quantity and quality [28], the cost of ignoring them will eventually outweigh the gain.

Recently, a number of novel GO-based semantic similarity measures for proteins have been proposed [25,30-33], employing various strategies. Future work will include the evaluation of these novel measures as well as investigating the relationship between gene products semantic similarity and other protein aspects, such as Pfam and Enzyme Commission classification.

## Methods

### Dataset

For our evaluation strategy we needed protein data (sequences), GO data (terms and graph structure) and annotation data (protein-term relationships). To that end, we built a local database that integrates the UniProt database, the GO database, and the GOA-UniProt [34] database, from the respective releases of February 2007. We calculated the IC of each GO term in our database, according to Resnik [5]:

IC(c)=−log⁡p(c)

where *p*(*c*) is the probability of usage of the term in the corpus, which in our case corresponds to the frequency with which the term is annotated. To obtain this frequency, we first count for each term the number of distinct proteins annotated to it or one of its descendent terms, and then divide that number by the total number of annotations within the corresponding GO type. After obtaining the IC from equation 1, we uniformize it by dividing by the scale maximum (so as to obtain a value in a 0-1 scale). The final expression for the uniform IC is:

$$IC_{U}\left(c\right)=\frac{IC\left(c\right)}{\log_{2}N}$$

with *N* being the total number of annotations within the corresponding GO type [35].

A subset of 22,067 Swiss-Prot proteins was selected from the database, consisting of proteins annotated to at least one molecular function GO term of IC 65% or higher. This criterion ensures that poorly annotated proteins (*i.e.* those with only very generic terms) are discarded, which would otherwise bias the semantic similarity results. The exact value of 65% was chosen as a compromise between computational time and representativity of the dataset (most of the poorly annotated proteins would probably be excluded at a lower cut-off).

An all-against-all BLAST search was performed with a threshold e-value of 10^−4^, resulting in a final (full) dataset of 618,146 distinct protein pairs. The e-value threshold ensures that the alignments considered are statistically significant.

To evaluate the influence of electronic annotations, a second subset of 4,608 proteins was selected using the above criteria, but where annotations with evidence codes IEA, NAS, NA and NR were discarded; this lead to a final (non-electronic) dataset of 49,480 protein pairs.

### Sequence similarity measures

Sequence similarity was computed according to two metrics: log reciprocal BLAST score (LRBS) and relative reciprocal BLAST score (RRBS). Given two proteins A and B, LRBS is simply given by the logarithm of the average of the BLAST bit scores resulting from BLASTing A against B and B against A:

$$LRBS\left(A,B\right)=\log_{10}\left(\frac{BLAST_{bitscore}\left(A,B\right)+BLAST_{bitscore}\left(B,A\right)}{2}\right)$$

This average is used to compensate for the fact that the BLAST bit scores are not symmetric (*i.e. BLAST_bitscore_*(*A*,*B*) ≠ *BLAST_bitscore_*(*B*,*A*)). Symmetry is a necessary property for all similarity measures in our evaluation; otherwise each protein pair could not be represented by a single data point. Because we wanted to test a second sequence similarity metric that was independent of sequence length, we developed the RRBS measure. Our goal was to have a metric analogous to sequence identity, but taking amino acid substitutions into account. Given two proteins A and B, RRBS is calculated by dividing the sum of their reciprocal BLAST bit scores by the sum of their self-BLAST bit scores:

$$RRBS\left(A,B\right)=\frac{BLAST_{bitscore}\left(A,B\right)+BLAST_{bitscore}\left(B,A\right)}{BLAST_{bitscore}\left(A,A\right)+BLAST_{bitscore}\left(B,B\right)}$$

Rather than count the number of equal amino acids and divide that by the total length of the alignment (sequence identity), this measure quantifies the whole alignment and divides that by the quantification of the perfect self-alignment (in both directions to ensure symmetry).

### Semantic similarity measures

A total of fourteen approaches to semantic similarity were tested, corresponding to four distinct approaches (GraSM, Average, Maximum and BMA) to each of the three ‘classic’ term semantic similarity measures: Resnik's [5], Lin's [6], and Jiang and Conrath's [7]; plus two graph-based measures: *simUI*[13] and *simGIC*[14].

#### 0.0.1 Term semantic similarity

The three term similarity measures were implemented as described by their authors. Given two terms *c*_1_ and *c*_2_ and their most informative common ancestor *c_A_*, Resnik's measure is given by [5]:

$$sim_{Res}\left(c_{1},c_{2}\right)=IC\left(c_{A}\right)$$

Lin's measure is given by [6]:

$$sim_{Lin}\left(c_{1},c_{2}\right)=\frac{2\times IC\left(c_{A}\right)}{IC\left(c_{1}\right)+IC\left(c_{2}\right)}$$

and Jiang and Conrath's similarity measure was derived from the distance measure as suggested by the authors [7], leading to the expression:

$$sim_{JC}\left(c_{1},c_{2}\right)=1+IC\left(c_{A}\right)-\frac{\left(IC\left(c_{1}\right)+IC\left(c_{2}\right)\right)}{2}$$

Since uniform IC values were used, all three similarity measures produced also uniform results (in a 0-1 scale). When the GraSM approach was used, the average IC of all disjoint common ancestors was considered instead of only that of the most informative [10]. As the influence of GraSM is independent of the method used to combine term similarities, and since it is a computationally intensive approach, it was applied to all three term measures but only using the BMA approach.

#### 0.0.2 Protein semantic similarity

Protein semantic similarity scores were calculated from the term similarity measures using three combination approaches: maximum (MAX), average (AVG) and best-match average (BMA) [11]. From each protein, the set of its direct annotations was obtained, and redundant annotations (*i.e*. those already inherited from an annotation to a descendent term) were excluded. Given two proteins A and B, with non-redundant sets of GO term annotation GO(A) and GO(B) respectively, the maximum approach is given by the maximum of the similarity between each term in GO(A) and each term in GO(B)

$$sim_{MAX}\left(A,B\right)=MAX_{t_{1}\in GO\left(A\right),t_{2}\in GO\left(B\right)}\left(sim\left(t_{1},t_{2}\right)\right)$$

the average approach is given by the average similarity between each term in GO(A) and each term in GO(B):

$$sim_{AVG}\left(A,B\right)=AVG_{t_{1}\in GO\left(A\right),t_{2}\in GO\left(B\right)}\left(sim\left(t_{1},t_{2}\right)\right)$$

and the best-match average approach is given by the average similarity between each term in GO(A) and its most similar term in GO(B), averaged with its reciprocal to obtain a symmetric score:

$$sim_{BMA}\left(A,B\right)=\frac{AVG_{t_{1}}\left(MAX_{t_{2}}sim\left(t_{1},t_{2}\right)\right)+AVG_{t_{2}}\left(MAX_{t_{1}}sim\left(t_{1},t_{2}\right)\right)}{2},t_{1}\in GO\left(A\right),t_{2}\in GO\left(B\right)$$

Protein semantic similarity was also calculated using the graph-based measures *simUI*[13] and *simGIC*[14]. In the case of these measures, for each protein, we obtain the extended set of its annotations, including direct annotations and all their ancestral terms up to the root node, which corresponds to a sub-graph of GO. Given two proteins A and B, with extended sets of GO term annotations GO(A) and GO(B) respectively, *simUI* is given by the number of terms in the intersection of GO(A) with GO(B) divided by the number of terms in their union [13]:

$$simUI\left(A,B\right)=\frac{COUNT_{t\in \left\{GO\left(A\right)\cap GO\left(B\right)\right\}}}{COUNT_{t\in \left\{GO\left(A\right)\cup GO\left(B\right)\right\}}}$$

whereas *simGIC* is given by the sum of the IC of each term in the intersection of GO(A) with GO(B) divided by the sum of the IC of each term in their union:

$$simGIC\left(A,B\right)=\frac{\sum_{t\in \left\{GO\left(A\right)\cap GO\left(B\right)\right\}}IC\left(t\right)}{\sum_{t\in \left\{GO\left(A\right)\cup GO\left(B\right)\right\}}IC\left(t\right)}$$

### Data processing and modelling

The raw semantic similarity *vs.* sequence similarity results consist of a high number of scattered data points, making it impossible to discern a pattern. This is expected since cases of functionally similar proteins with unrelated sequences, and vice-versa, are well known to occur, if not frequently. However, as we are interested in studying the global pattern, semantic similarity values were averaged over sequence similarity intervals (separately for each of the sequence similarity metrics). Intervals were taken with constant number of data points to ensure all intervals are equally representative, and for each interval the average values of sequence and semantic similarity were computed. In the full dataset each interval contains 5,000 points, and in the non-electronic dataset each interval contains 1,000 points.

The behaviour of the averaged semantic similarity *vs*. sequence similarity results was modelled using Normal cumulative distribution functions (*N_CDF_*), *i.e*. error functions, transformed by scale and translation parameters. Function parameters were fitted to the results using the least squares method and the Newton optimisation algorithm. Initially the results were modelled by a single *N_CDF_*, of the form [14]:

$$Semantic_{sim}=a+b\times N_{CDF}\left(Sequence_{sim},\mu ,\sigma \right)=\frac{1}{2}\left[a+b\times erf\left(\frac{Sequence_{sim}-\mu }{\sigma \sqrt{2}}\right)\right]$$

where *a* is a translation factor to account for the fact that the minimum (averaged) semantic similarity values are greater than zero; *b* is a scale factor to account for the range of the (averaged) semantic similarity values being different than 0-1; μ is the Normal mean, which corresponds to the inflection point of the curve; σ is the Normal standard deviation, which reflects for the spread of the curve along the sequence similarity axis; and *er f* stands for the error function. All parameters (*a*, *b*, μ and σ) were adjusted to fit the data. However, we found that for the full dataset, a single *N_CDF_* could not suitably describe all the aspects of the behaviour of the data, namely the evident bimodal-like behaviour, *i.e* the fact that there are two distinct zones where semantic similarity visibly increases with sequence similarity, separated by a transition zone where it is approximately constant. To account for this behaviour, we added a second *N_CDF_* to the modelling function, resulting in:

$$Semantic_{sim}=a+b\times N_{CDF}\left(Sequence_{sim},\mu_{1},\sigma_{1}\right)+c\times N_{CDF}\left(Sequence_{sim},\mu_{2},\sigma_{2}\right)$$

Here *b*, μ_1_ and σ_1_ are the scale, mean and standard deviation parameters for the first *N_CDF_*, while *c*, μ_2_ and σ_2_ are the corresponding parameters for the second *N_CDF_*.

**Figure 7:**
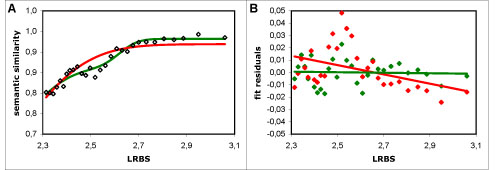
**Bimodal vs. unimodal fit to the semantic similarity results.** A - Semantic similarity (Resnik's measure with the BMA approach) *vs*. LRBS sequence similarity: black points - averaged results; red line - unimodal modelling function; green line - bimodal modelling function. B - Fit residuals of the bimodal and unimodal modelling functions *vs*. LRBS sequence similarity: red points - fit residuals of the unimodal modelling function; red line - corresponding linear trendline; green points - fit residuals of the bimodal modelling function; green line - corresponding linear trendline. It is clear that the unimodal modelling function does not describe the behaviour of the results accurately, since the fit residuals are unevenly distributed (as reflected by the negative slope of the trendline); whereas the bimodal modelling function shows evenly distributed residuals (with a nearly horizontal trendline at 0).

The addition of the second *N_CDF_* visibly improves the quality of the model (Figure 7A), reducing the sum of the squared residuals by 14-27%. Moreover, the dispersion of the residuals becomes centred, whereas with a single *N_CDF_* it was noticeably skewed (Figure 7B).

For the measures using the average combination approach there was an evident decreasing behaviour for high sequence similarity values, which is impossible to model with a monotonically increasing function like *N_CDF_*. To account for this behaviour, we added a linear component (*d* × *Sequence_sim_*) to the modelling function 14, since the decrease was approximately linear. This component was added for the whole sequence similarity range, and not only for the decreasing portion, since the other parameters of the modelling function are able to compensate for its presence and model the behaviour of the data outside of that portion.

The results using the non-electronic dataset were modelled using only a single *N_CDF_* 13, as there was no visible sign of bimodality.

### Evaluation parameter

The main parameter we used to evaluate the measures was resolution, *i.e* the range of the averaged semantic similarity results. Resolution was calculated by the sum of the two scale parameters for the results in the full dataset (since they are modelled by two *N_CDF_*s), and is simply given by the scale parameter for the results in the non-electronic dataset with the LRBS sequence similarity measure. However, for the results in the non-electronic dataset with the RRBS sequence similarity measure, resolution cannot be calculated from the scale parameter because for the majority of the measures the fitted *N_CDF_* is not contained in the range of the results, and consequently the scale parameter is greater than 1. Therefore, in the case of these results, resolution was calculated as the difference between the maximum and the minimum of the averaged semantic similarity values.

## Authors' contributions

This works was a collaboration with equal contribution from CP and DF, under the supervision of AOF, AEF and FC. HB provided the BLAST results. All authors reviewed the final manuscript.

## Competing interests

The authors declare they have no competing interests.
